# Spheroacanthocytes secondary to novel tyrosine kinase inhibitors

**DOI:** 10.1002/jha2.104

**Published:** 2020-09-16

**Authors:** Michael Ashby, Michael Sze Yuan Low

**Affiliations:** ^1^ Monash Haematology Monash Medical Centre Victoria Australia

A 64‐year‐old male with ALK‐positive nonsmall cell lung carcinoma on oral therapy with alectinib presented with pleural effusion. Full blood examination revealed hemoglobin of 90 g/L, white blood cell count 12.4 × 10^9^/L, and platelet count 372 × 10^9^/L. Peripheral blood film displayed marked acanthocytes and spheroacanthocytes (Figure 1: spherocytes (black arrow) and spheroacanthocytes (red arrow)) with negative hemolytic indices including normal reticulocyte count, 75 × 10^9^/L, high haptoglobin 2.18 g/L (range 0.36‐1.95), mildly elevated lactate dehydrogenase 317 U/L (range 120‐250), normal bilirubin 16 mcmol/L (range 0‐20), and a negative poly‐specific direct antiglobulin test. Liver function tests were essentially normal: alkaline phosphatase mildly elevated 119 U/L (30‐110), gamma‐glutamyl transferase 15 U/L (5‐50), and alanine aminotransferase 23 U/L (5‐40). Flow cytometry demonstrated reduced eosin‐5‐maleimide binding, with mean channel fluorescence of 5.6 (control average MCF 12.87). Alectinib is a novel tyrosine kinase inhibitor approved for use in patients with nonsmall cell lung cancer. Alectinib is known to cause anemia but the striking acanthocytes and spheroacanthocytes have only been reported in a few case series. Spheroacanthocytes are commonly associated with end‐stage liver disease; however, hematopathologists should recognize alectinib as an alternate cause for acanthocytes and spheroacanthocytes on a peripheral blood film.

**FIGURE 1:**
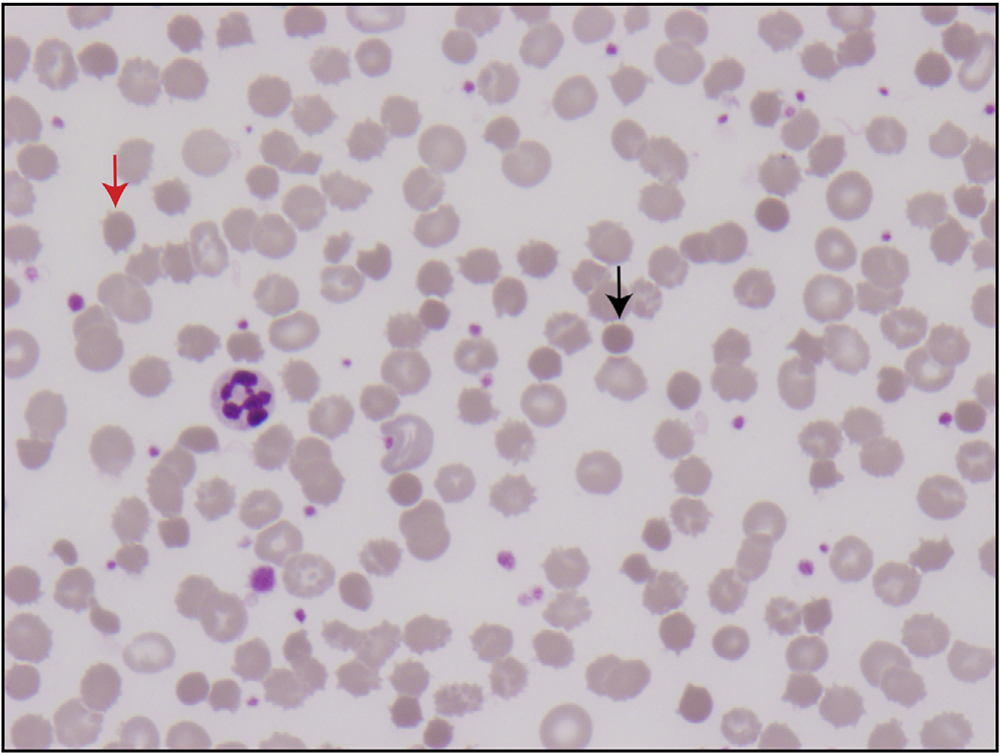
Spherocytes (black arrow) and spheroacanthocytes (red arrow)

## CONFLICT OF INTEREST

The authors declare no conflict of interest.

## CONSENT TO PARTICIPATE

The patient presented has given consent for his case to be published in a de‐identified manner.

